# Risk Factors for Multidrug-Resistant Gram-Negative Bacteria Carriage upon Admission to the Intensive Care Unit

**DOI:** 10.3390/ijerph19031039

**Published:** 2022-01-18

**Authors:** Nicolás Francisco Fernández-Martínez, Sheila Cárcel-Fernández, Carmen De la Fuente-Martos, Rafael Ruiz-Montero, Bernardo R. Guzmán-Herrador, Rafael León-López, Francisco Javier Gómez, Julia Guzmán-Puche, Luis Martínez-Martínez, Inmaculada Salcedo-Leal

**Affiliations:** 1 Preventive Medicine and Public Health Unit, Reina Sofía University Hospital, 14004 Córdoba, Spain; nicolasf.fernandez.sspa@juntadeandalucia.es (N.F.F.-M.); berguzherr@gmail.com (B.R.G.-H.); minmaculada.salcedo.sspa@juntadeandalucia.es (I.S.-L.); 2 Maimonides Biomedical Research Institute of Cordoba (IMIBIC), 14004 Córdoba, Spain; sheilacarfer@gmail.com (S.C.-F.); carmen.fuente.sspa@juntadeandalucia.es (C.D.l.F.-M.); RAFAEL.LEON.LOP@hotmail.com (R.L.-L.); juliam.guzman.sspa@juntadeandalucia.es (J.G.-P.); luis.martinez.martinez.sspa@juntadeandalucia.es (L.M.-M.); 3 Intensive Care Unit, Reina Sofía University Hospital, 14004 Córdoba, Spain; 4 Department of Medicine, University of Granada, 18011 Granada, Spain; fgomez@ugr.es; 5 Department of Medical and Surgical Sciences, University of Córdoba, 14004 Córdoba, Spain; 6 Microbiology Unit, Reina Sofía University Hospital, 14004 Córdoba, Spain; 7 Centro de Investigación Biomédica en Red de Enfermedades Infecciosas (CIBERINFEC), Carlos III Research Institute, 28029 Majadahonda, Spain; 8 Department of Agricultural Chemistry, Soil Science and Microbiology, University of Córdoba, 14004 Córdoba, Spain

**Keywords:** antibacterial drug resistance, critical care, infection control, risk factors, gram negative bacteria, epidemiology

## Abstract

Multidrug-resistant Gram-negative bacteria (MDR-GNB) are microorganisms that have acquired resistance to extended-spectrum antibacterials and constitute an emerging threat to public health. Although carriers are an important source of transmission in healthcare settings, data about risk factors for MDR-GNB carriage are limited. Therefore, we aimed to identify risk factors for MDR-GNB carriage upon intensive care unit (ICU) admission and to optimise screening strategies. We conducted a case–control study. Admissions of adult patients to the ICU of a 1000-bed hospital during a year were included. We collected sociodemographic, clinical and microbiological data and performed a multivariate logistic regression model. A total of 1342 patients resulted in 1476 episodes of ICU admission, 91 (6.2%) of whom harboured MDR-GNB (38.5% women; median age 63.9 years). The most frequently isolated pathogens were *Escherichia coli* (57%) and *Klebsiella pneumoniae* (16%). The most frequent resistance mechanism was production of extended-spectrum beta lactamases. MDR-GNB carriage was associated to liver cirrhosis (OR 6.54, 95% CI 2.17–19.17), previous MDR-GNB carriage (OR 5.34, 1.55–16.60), digestive surgery (OR 2.83, 1.29–5.89) and length of hospital stay (OR 1.01 per day, 1.00–1.03). Several risk factors for MDR-GNB carriage upon admission to a high-risk setting were identified; the main comorbidity was liver cirrhosis.

## 1. Introduction

Multidrug-resistant Gram-negative bacteria (MDR-GNB) are microorganisms that have acquired, through a variety of mechanisms, resistance to extended-spectrum antibacterials, for instance third-generation cephalosporins, fluoroquinolones and carbapenems [1]. The gut microbiota is the main reservoir of MDR-GNB, which can act as opportunistic pathogens in patients at risk. The term healthcare-associated infections (HAIs) refers to infections where healthcare settings are the source of transmission; among them, HAIs in critically ill patients are of particular concern. Colonisation by these bacteria in asymptomatic carriers is considered a potential source of cross-transmission between patients. Furthermore, being colonised substantially increases the risk of MDR-GNB infections, characterised by poor outcomes due to treatment failure: increased morbimortality and hospital length of stay, as well as higher health care costs [2].

The frequency of HAIs caused by MDR-GNB has increased dramatically [3] and constitutes an emerging threat to public health worldwide [4]. In the European context, MDR-*Klebsiella pneumoniae*, MDR-*Acinetobacter baumannii* and MDR-*Escherichia coli* have been described as the main emerging MDR-GNB: in 2019, more than half of the E. coli isolates and more than a third of the K. pneumoniae isolates were resistant to at least one antimicrobial group under surveillance. Multidrug resistance is not homogeneous in the region, with countries in Southern and Eastern Europe showing higher percentages of resistant bacteria [5].

An essential preventive measure included in several clinical practice guidelines is the systematic screening of patients at risk, which allows for the early identification of carriers [6]. Several European countries are already implementing this strategy, although it is still under review because risk factors, along with the relevance of each of them, are not well characterised. Disparities exist between organisations such as the European Centre for Disease Prevention and Control [7], the Society for Healthcare Epidemiology of America [8] and the Spanish Ministry of Health (“Zero Resistance” program, leaded by the Spanish Society of Intensive Care) [9]. The scientific literature suggests that advanced age, disability, certain comorbidities, previous antibacterial therapy, cancer chemotherapy, previous gastrointestinal surgery, history of stay in healthcare settings or long-term care facilities and previous detection of MDR-GNB, would increase the risk of colonisation [10,11]; nonetheless, evidence remains scarce. As a control measure resulting from a long-lasting outbreak of KPC-3-producing *Klebsiella pneumoniae* (ST512) at our hospital from 2012 to 2015 [12], it was decided to collect a screening rectal swab from every patient upon intensive care unit (ICU) admission to prevent the nosocomial spread of MDR-GNB.

This study targets two hurdles: limited data about the frequency and risk factors for carriage of MDR-GNB and suboptimal use of resources due to routine screening to all patients (high costs, poor compliance with infection prevention and control measures and unnecessary workload for laboratory staff).

Our main objective was to identify risk factors for MDR-GNB carriage to better target the screening strategy upon ICU admission for patients at risk. We also describe the prevalence of MDR-GNB carriage and the pathogens involved.

The study hypothesis was that previous antibacterial therapy and history of stay in healthcare settings or long-term care facilities are associated with a higher risk of carrying MDR-GNB.

## 2. Materials and Methods

### 2.1. Study Design

A non-matched, hospital-based, case-control study was conducted to investigate which factors were associated with an increased risk of MDR-GNB carriage upon ICU admission. Cases were defined as episodes of admission to the ICU in which patients had a positive result to any of the MDR-GNB screened upon admission in the ICU. Prevalent cases, which were excluded from the analysis to avoid Neyman bias, were defined as cases with detection (in any clinical or screening sample) of carbapenemase-producing GNB in the previous 24 months, or MDR-GNB other than carbapenemase-producing in the previous 12 months. This definition follows criteria from national and international organisations [7,13], adapted to regional guidelines. Controls were defined as episodes of admission to the ICU for patients showing detection of non-MDR GNB, or a negative result in such screening.

### 2.2. Study Population

The study population was all critically ill patients admitted to the adult ICU of Reina Sofía University Hospital, a tertiary 1000-bed hospital, during the study period. The adult ICU has 42 beds, six modules (coronary and cardiothoracic, transplantation, neurotrauma and 3 medical intensive modules) and has an average of 1500 admissions per year. The main MDR-GNB causing HAIs in the ICU over the past years have been *Escherichia coli*, *Enterobacter cloacae*, *Pseudomonas aeruginosa* and *Klebsiella* sp. (notably *K. pneumoniae*). Extended spectrum betalactamases (ESBL) are, by far, the most frequent mechanism of resistance, but carbapenem-resistant Enterobacterales (CRE) are on the rise.

Eligibility criteria and study period. All adult patients (aged ≥16 years) admitted to the ICU from 1 November 2018 to 31 October 2019 were eligible.

Variables. The independent variables were: (a) sociodemographic: age, sex and stay in congregate settings (e.g., nursing homes and prisons); (b) clinical: APACHE II score, referring ward, previous carriage of MDR-GNB, chronic comorbidities (cystic fibrosis, bronchiectasis, COPD, chronic ulcers, liver cirrhosis, immunodeficiency, cancer, neutropenia, type 1 diabetes mellitus and type 2 diabetes mellitus), previous antibacterial therapy (defined by at least seven days in the last month), gastrointestinal surgery in the last year, gastrointestinal endoscopy in the last year, history of previous transplantation (type), cancer chemotherapy, dialysis, presence of open wounds, biliary drainage, number and length of hospital admissions in the last 12 months (including the actual hospital admission prior to ICU admission), hospital admission date and ICU admission date; and (c) microbiological: microorganism(s) isolated and mechanism(s) of antimicrobial resistance. The dependent variable was the result of the MDR-GNB screening. Variables included were qualitative, with the exception of age, APACHE II score, number and length of hospital admissions, which were quantitative.

This study was devised following the Strengthening the Reporting of Observational Studies in Epidemiology (STROBE) recommendations [14] (see Supplementary Table S3).

### 2.3. Data Collection

Patient information was obtained through direct interview with the patient or the responsible physician and from the patient’s clinical records. Rectal swab specimens were collected in all study participants within the first 24 h of ICU admission to assess bacterial colonisation. Swabs collected were placed in sterile round bottom tubes containing 1 mL sterile Copan transport medium and immediately transferred to the Clinical Microbiology Laboratory for analysis. Details on the analysis can be found in Supplementary Text S1.

We considered MDR-GNB as that with mechanisms of resistance requiring contact precautions for infection control and followed international expert criteria [15] adapted to regional guidelines; essentially, genotypic detection of relevant beta-lactamases (ESBL production or ampC derepression) or any carbapenemases (Ambler classes A, B or D). The exceptions to this rule (MDR *P. aeruginosa*, *A. baumannii* and *S. maltophilia*) can be consulted elsewhere [16].

### 2.4. Statistical Analysis

In the descriptive analysis, qualitative variables were expressed as absolute numbers and their relative frequencies, whereas quantitative variables, tested for normal distribution by the Shapiro–Wilk test, were expressed as mean and standard deviation (SD) if normally distributed, or as median and interquartile range (IQR) if not. In the bivariate analysis, Pearson’s chi-square was used to compare categorical variables, Student’s *T*-test was used for mean comparison in quantitative variables and Wald’s test was used to obtain crude (non-adjusted) estimators for quantitative variables. Non-parametric tests were used when appropriate.

We performed a backward, conditional, 10-fold cross-validated stepwise multivariate logistic regression model to preselect predictive variables. Overall goodness of fit was analysed by Akiake’s Information Criteria (AIC). These preselected variables, along with those associated with MDR-GNB in the bivariate analysis with a *p*-value <0.30, as well as key sociodemographic variables, were included in a backward, multivariate logistic regression model. Collinearity of the model was measured by the variance inflation factors, setting the threshold of non-collinearity at <2.5. Linear relation between quantitative independent variables and the dependent variable was examined by the added-variable plot. Calibration of the model was assessed by Hosmer–Lemeshow test. Discrimination of the model was assessed by receiver-operator curve (ROC) characteristics with 95% confidence interval (CI). Significance of the regression coefficients was assessed by Wald’s test.

First, we built an explanatory model maximising accuracy to achieve the primary objective. Then, we prioritszed sensitivity, by lowering the threshold for positivity from 0.5 to 0.05, in a pragmatic approach to achieve the secondary objectives. Finally, we compared the performance of both models, through sensitivity, specificity, negative predictive value (NPV), positive predictive value (PPV) and accuracy for different breakpoints.

All statistical tests were two-sided, and variables with a *p*-value of 0.05 or less were considered significant. The analyses were performed using R software version 4.0.3 (R Foundation for Statistical Computing, Vienna, Austria).

## 3. Results

**Figure 1:**
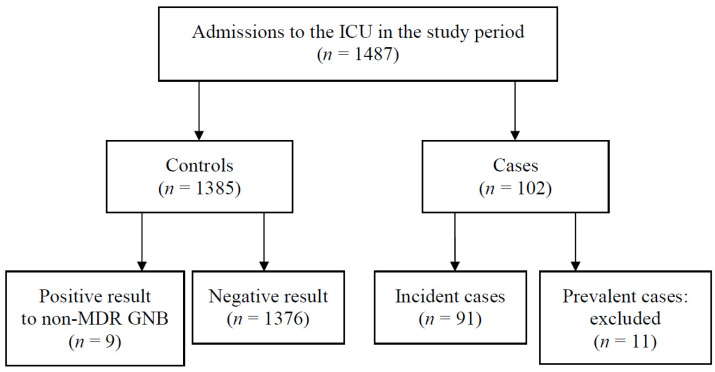
Distribution of the study participants.

From 1 November 2018 to 31 October 2019, there were 1342 patients admitted to the ICU, resulting in 1487 episodes of ICU admission. Of these 1487 episodes, 102 (6.86%) were identified as cases, of which 91 (89.22%) were incident cases. Only 11 subjects were classified as prevalent cases and consequently excluded from the analysis; the characteristics of these patients are reported in Supplementary Table S1. Among the 1385 (93.14%) controls, nine showed a positive result to non-MDR GNB and the rest had a negative result (Figure 1).

### 3.1. Univariate and Bivariate Analysis

Of the 1476 cases (91) and controls (1385) finally included, 491 were female (33.27%) and the median age was 63 years. Baseline characteristics are shown in Table 1. Cases and controls were similar in terms of age, severity and most comorbidities. Significant differences were observed between both groups. Previous MDR-GNB carriage, previous antibacterial therapy, liver cirrhosis, previous gastrointestinal endoscopy, digestive surgery in the last year and prolonged length of hospital stay in the last year were more frequent in cases.

Regarding the referring ward, cases were more frequently referred from hepatology, general and digestive surgery, whereas controls came more frequently from cardiovascular surgery and the emergency department. We show previous antibacterial therapy administered to cases and controls in Supplementary Table S2. Additionally, female sex, cancer, neutropenia and solid organ transplantation were also more common among cases; however, these differences did not reach statistical significance.

Pathogens identified among cases are displayed in Table 2. The GNB most frequently isolated were *Escherichia coli* (*n* = 58, 57%) and *Klebsiella pneumoniae* (*n* = 16, 16%), followed by *Citrobacter freundii* and *Klebsiella aerogenes*. In terms of antimicrobial resistance mechanisms, ESBL-producing *E. coli* and other ESBL-producers were the most common (*n* = 77, 76%) MDR-GNB, followed by derepressed ampC-producers (15, 15%. Other mechanisms such as carbapenemase production (*n* = 7, 7%, with KPC-producing *K. pneumoniae* accounting for almost half of this group) were unusual.

### 3.2. Multivariate Analysis

Multivariate analysis (Table 3) showed that liver cirrhosis (OR 6.54, 95% CI 2.17–19.17, *p* < 0.01), previous MDR-GNB carriage (OR 5.34, 95% CI 1.55–16.60, *p* < 0.01), digestive surgery in the last year (OR 2.83, 95% CI 1.29–5.89, *p* < 0.01) and length of hospital stay in the last year (OR 1.01 per each additional day, 95% CI 1.00–1.03, *p* 0.03) were independent risk factors for MDR-GNB carriage upon ICU admission. Our data also suggest that previous gastrointestinal endoscopy, patients classified as non-emergency surgical and medical by the APACHE system, as well as previous antibacterial therapy may confer a higher risk of MDR-GNB carriage, while cancer chemotherapy might be associated with a lower risk. Overall, the model showed a good calibration, (*p* > 0.05 in the Hosmer–Lemeshow test), and collinearity was low (variance inflation factors <2 for all included variables).

**Figure 2:**
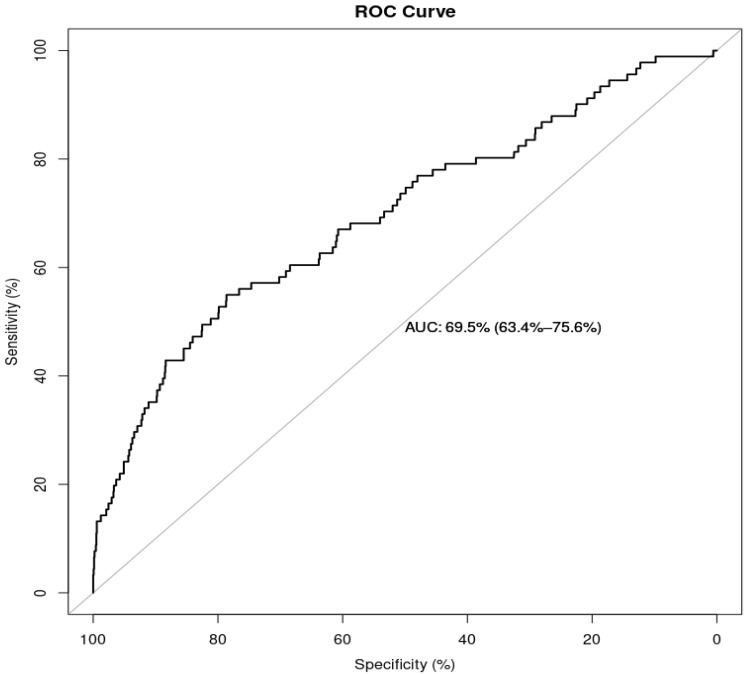
ROC (receiver operating characteristic) curve of the logistic regression model.

The ROC curve (Figure 2) could correctly discriminate up to 70% of cases and controls, with an area under the curve of 0.70 (95% CI 0.63–0.76). In an explanatory model with a breakpoint of 0.50 and prioritising accuracy: accuracy (94.0%), specificity (99.9%) and negative predictive value (94.1%) were high, while positive predictive value was acceptable (71.4%) but sensitivity was very low (5.5%). In a pragmatic model with a breakpoint of 0.05 and prioritising sensitivity, we achieved a 10-fold increase in sensitivity (62.6%) and a negative predictive value of 96.2%, with a decrease in specificity (62.4%) and a positive predictive value (9.9%). Despite testing additional cut-off points for the pragmatic model, there was no optimal breakpoint for positive and negative predictive values, due to low sensitivity and a pre-test prevalence of roughly 6.2%.

## 4. Discussion

In our study, 7% of screening cultures upon ICU admission were positive for MDR-GNB, mainly ESBL-producing *E. coli* and *K. pneumoniae*. These findings are consistent across studies of critically ill patients, although the frequency of rectal carriage of MDR bacteria reported in other ICUs is somewhat higher, with rates over 10% [10].

Our study has identified several risk factors independently associated with MDR-GNB carriage upon ICU admission. Interestingly, among them only one comorbidity showed a strong association–liver cirrhosis. Previous studies carried out in Spain have reported liver cirrhosis as a risk factor for MDR colonisation in critically ill patients [17]; in decompensated cirrhosis, norfloxacin prophylaxis and ascites may be the main factors related to colonisation upon admission and follow-up, respectively [18].

Liver cirrhosis can lead to carriage of MDR-bacteria through a plethora of immunological mechanisms grouped under the term, cirrhosis-associated immune dysfunction. The initial systemic inflammation, responsible for fibrosis and complications, such as portal hypertension, gradually switches to an immunodeficient state, characterised by exposure to gut microbial products that chronically stimulate innate immune cells, impairing their antimicrobial function in advanced disease [19]. However, other well-known conditions causing a higher risk of infection, as primary immunodeficiencies, solid organ and hematopoietic stem cell transplantation, end-stage renal disease requiring dialysis, and neutropenia were not identified as risk factors. In fact, we found that cancer chemotherapy might be linked to a lower risk but there is a potential of selection bias, since a proportion of patients undergoing cancer chemotherapy are less likely to be admitted to the ICU [20]. Besides immunological mechanisms, liver cirrhosis can promote bacterial infection via microbiome alterations and intestinal barrier dysfunctions [21].

Digestive disorders other than liver cirrhosis play a role, at least indirectly, in the carriage of MDR-GNB: digestive surgery in the last year was an independent risk factor, and previous gastrointestinal endoscopy, associated with a two-fold risk of carriage, was barely below the level of significance (*p* = 0.067). Surgery in the last year increases the risk of carriage with ESBL-producing bacteria in the ICU and upon hospital admission, and recent surgery that of KPC-producing *K. pneumoniae* carriage [22]. With regard to gastrointestinal endoscopy, a systematic review identified five outbreaks of carbapenem-resistant Enterobacterales (CRE) associated with duodenal endoscopy, one of which took place in a medical ICU [23], and upper gastrointestinal endoscopy has also been reported as a risk factor for CRE colonisation at ICU admission [24].

Prior stay in healthcare settings is a risk factor for acquisition of MDR-GNB [7]. Our study shows this relationship is dose-dependent: length of hospital stay in the last year was associated with MDR-GNB carriage upon ICU admission, increasing the risk by 1% per day of previous hospital stay. Similar results have been found for the risk of fecal carriage of MDR *E. coli* and MDR *K. pneumoniae* [25], and for the risk of infection by carbapenem-resistant GNB [26], particularly in critically ill patients [27].

Previous MDR-GNB carriage was an independent risk factor too, despite exclusion of prevalent cases. This finding, albeit expected, is concerning, since most MDR-GNB isolated upon ICU admission were ESBL producers, and long duration of carriage (>12 months) is usually reported for CRE [28,29,30]. Since 2018, hospitals in Andalusia, Spain, have substantially improved early assessment of previous MDR-GNB carriage and implementation of IPC measures after introducing HAM (Health Alert Monitoring). HAM [16] is an automated system that gives an alert at hospital admission for every patient with a history of prior detection of MDR bacteria (as a matter of fact, the experience has been so successful that HAM is also being used for SARS-CoV-2).

Misuse and overuse of antimicrobial agents is a key factor driving multidrug resistance [31]. Even though a higher rate of MDR-GNB carriage was observed in patients receiving previous antibacterial therapy, our data could not confirm this association. We used strict criteria in terms of extent of antibacterial exposure and resistance outcome, which are crucial for the epidemiological interpretation of resistance studies [32] and could explain the absence of statistical significance. For instance, patients who received antibacterial therapy during less than 7 days in the month prior to ICU admission were classified as not exposed, and carriers of *S. maltophilia* resistant to quinolones and colistin but susceptible to trimethoprim–sulfamethoxazole were classified as controls.

Certain settings, especially nursing homes, increase the risk of acquiring MDR-GNB [33]. Nevertheless, such association was not found in our study, since the proportion of patients with a history of stay in congregate settings was below 1%. A possible explanation is that patients from long-term care facilities, because of advanced age and dependence in activities of daily living, are less likely to benefit from critical care.

Unfortunately, it was not possible to develop a risk score for MDR-GNB carriage upon ICU admission. The pragmatic model yielded a sensitivity of 62.6% and a positive predictive value of 9.9%, which we deem insufficient for an adequate detection of carriers because underdiagnosis increases the likelihood of outbreaks by MDR-GNB. Remarkably, similar barriers for prediction have been reported for MDR bacteria colonisation or infection at ICU admission [34] and, more recently, for carriage at hospital admission [35], suggesting that many of the risk factors for MDR-GNB carriage are yet to be discovered.

Until such uncertainties are addressed, infection prevention and control measures are essential to reduce the spread of MDR-GNB. These include, but are not limited to: (a) compliance with standard and contact precautions [36,37] (i.e., hand hygiene and appropriate use of gloves and gowns); (b) active microbiological surveillance [38]; (c) reduction in device use to prevent catheter–associated infection and ventilator–associated pneumonia [39]; (d) adequate cleaning and disinfection of environmental surfaces [40] and reusable devices [41], especially duodenoscopes [42]; (e) antimicrobial stewardship, with particular emphasis on surgical antimicrobial prophylaxis [43]; and (f) selective digestive decontamination [44], with promising results that should, however, be interpreted with caution [45]. This will not be an easy endeavour, as the challenges to tackling antimicrobial resistance will be unprecedented in the aftermath of the COVID-19 pandemic, due to changes in antimicrobial usage and availability [46].

This study has some limitations. Since our findings are based on the analysis of fewer than one hundred cases, certain risk factors might have not been identified due to a limited statistical power. Hospital controls were selected, who might share risk exposures with cases and often present risk factors at a higher frequency than the general population; nonetheless, this study focuses on MDR-GNB carriage in critical patients, who are definitely not representative of the general population. Furthermore, not matching cases and controls increases the risk of confusion bias but the robust multivariate analysis addresses this issue. Although MDR-GNB carriage was evaluated according to the screening result, we did not examine its meaning: was it colonisation and infection or solely colonisation? This is probably the main limitation of this study, since risk factors for colonisation and infection by MDR organisms might be different. We cannot rule out this possibility, as the available literature focuses on infection [47,48,49] and high-quality, up-to-date evidence related to colonisation remains scarce [50,51]. Finally, our results may not be extrapolated to other clinical settings, as the profile of critically ill patients admitted to our ICU may differ from that of smaller ICUs or those without transplantation modules. Furthermore, the MDR-GNB epidemiology depends on the local context, showing a high variability between countries [5].

Multidrug resistance should be seen as a problem that needs to be addressed long before a patient is admitted to an ICU or even to a hospital. Its approach requires coordination across different sectors from a One Health perspective [52]; in the health sector, involvement of both primary care and hospital care is needed, in order to achieve visible results in the medium term.

## 5. Conclusions

In conclusion, our study provides insight into the epidemiology of MDR-GNB carriage upon ICU admission. Those with liver cirrhosis are at highest risk and must therefore be systematically screened. Digestive surgery in the last year, prolonged hospital stay and previous carriage of MDR-GNB must also be considered. Despite having identified a number of critical risk factors and a relatively low carriage rate, we cannot discourage universal screening, given the potential impact of unidentified carriers in high-risk settings.

For a better understanding of the profile of carriers of these “superbugs”, further research is warranted: larger, prospective studies collecting comprehensive data on exposure (comorbidities, type and duration of antimicrobial therapy and device use, invasive procedures and prior stay in healthcare settings) and outcomes (colonisation versus infection, epidemiological significance and duration of carriage) will help find the missing pieces of the multidrug resistance puzzle.

## Figures and Tables

**Table 1:** Baseline characteristics of cases and controls upon ICU admission.

Characteristic	Cases *n* = 91 (6.2%)	Controls *n* = 1385 (93.8%)	*p* Value
Age (years), median (IQR)	63.87 (52.94, 74.10)	63.16 (52.60, 73.14)	0.933 ^1^
Sex			0.331 ^2^
Female	35 (38.5%)	456 (32.9%)	
Male	56 (61.5%)	929 (67.1%)	
Stay in congregate settings	0	7 (0.5%)	1 ^3^
Nursing home	0	4 (0.3%)	
Prison	0	2 (0.1%)	
Other	0	1 (0.1%)	
Referring ward			<0.001 *^4^
Cardiology	24 (26.4%)	361 (26.1%)	
Cardiovascular surgery	9 (9.9%)	283 (20.4%)	
General & Digestive surgery	7 (7.7%)	35 (2.5%)	
Emergency Department	19 (20.9%)	394 (28.4%)	
Hepatology	12 (13.2%)	63 (4.5%)	
Pulmonology	4 (4.4%)	69 (5.0%)	
Other	16 (17.6%)	180 (13.0%)	
APACHE classification system			0.119 ^4^
Medical	73 (80.2%)	977 (70.5%)	
Non-emergency surgical	13 (14.3%)	261 (18.8%)	
Emergency surgical	5 (5.5%)	147 (10.6%)	
Previous hospital admissions	44 (48.4%)	609 (44.0%)	0.480 ^2^
Previous hospital admissions (number), median (IQR)	1 (1, 2)	1 (1, 2)	0.311 ^1^
Length of hospital stay in the last year (days), median (IQR)	5 (0, 16.50)	2 (0, 7)	0.001 *^1^
Previous MDR-GNB carriage	6 (6.6%)	13 (0.9%)	0.001 *^3^
Cancer chemotherapy	2 (2.2%)	46 (3.3%)	0.844 ^3^
Dialysis	1 (1.1%)	18 (1.3%)	1 ^3^
Previous antibacterial therapy	15 (16.5%)	57 (4.1%)	<0.001 *^3^
Previous therapy with third-, fourth- or fifth-generation cephalosporins	2 (2.2%)	10 (0.7%)	0.332 ^3^
Previous therapy with carbapenems	2 (2.2%)	6 (0.4%)	0.165 ^3^
Cystic fibrosis	0	15 (1.1%)	0.766 ^3^
Bronchiectasis	1 (1.1%)	18 (1.3%)	1 ^3^
COPD	9 (9.9%)	109 (7.9%)	0.625 ^2^
Chronic ulcers	0	0	-
Liver cirrhosis	8 (8.8%)	46 (3.3%)	0.030 *^3^
Immunodeficiency	1 (1.1%)	12 (0.9%)	1 ^3^
Cancer	12 (13.2%)	131 (9.5%)	0.326 ^2^
Neutropenia	6 (6.6%)	35 (2.5%)	0.073 ^3^
Type 1 diabetes	2 (2.2%)	20 (1.4)	0.796 ^3^
Type 2 diabetes	21 (23.1%)	322 (23.2%)	0.928 ^2^
Pressure ulcers	1 (1.1%)	10 (0.7%)	1 ^3^
Digestive surgery in the last year	15 (16.5%)	50 (3.6%)	<0.001 *^3^
Previous gastrointestinal endoscopy	11 (12.1%)	54 (3.9%)	0.001 *^4^
Upper	7 (7.7%)	26 (1.9%)	
Lower	4 (4.4%)	29 (2.1%)	
APACHE II score (points), mean (SD)	17.76 (9.57)	18.41 (7.51)	0.528 ^5^
Solid organ transplantation	10 (11.0%)	123 (8.9%)	0.623 ^2^
Hematopoietic stem cell transplantation	0	4 (0.3%)	1 ^3^
Biliary drainage	1 (1.1%)	1 (0.1%)	0.239 ^3^

APACHE: Acute Physiology and Chronic Health Evaluation, COPD: chronic obstructive pulmonary disease, IQR: interquartile range, MDR-GNB: multidrug-resistant Gram-negative bacteria, SD: standard deviation. * *p*-value < 0.05 of Mann–Whitney U test ^1^, Pearson’s chi-square test ^2^, Fisher’s exact test ^3^, ANOVA’s test ^4^ or Student’s *t*-test ^5^, when appropriate.

**Table 2:** MDR-GNB identified in screening upon ICU admission.

MDR-GNB	Mechanism(s) of Resistance	*n*	% (Same Species)	% (Overall)
*Escherichia coli*	ESBL production	56	96.6	55.4
	IMP production	1	1.7	1.0
	OXA-48 production	1	1.7	1.0
*Klebsiella pneumoniae*	ESBL production	13	81.3	12.9
	IMP production	1	6.3	1.0
	KPC production	1	6.3	1.0
	OXA-48 production	1	6.3	1.0
*Klebsiella aerogenes*	AmpC derepression	4	80	4.0
	ESBL production	1	20	1.0
*Klebsiella oxytoca*	ESBL production	4	100	4.0
*Citrobacter freundii*	AmpC derepression	7	100	6.9
*Enterobacter cloacae*	AmpC derepression	4	66.7	4.0
	ESBL production	1	16.7	1.0
	OXA-48 production	1	16.7	1.0
*Proteus mirabilis*	ESBL production	2	66.7	2.0
	OXA-48 production	1	33.3	1.0
*Pseudomonas aeruginosa*	Other *	2	100	2.0
Total	-	101	-	100

ESBL: extended spectrum beta lactamases, MDR-GNB: multidrug-resistant Gram-negative bacteria. * According to international criteria adapted to regional guidelines, as defined previously.

**Table 3:** Risk factors for MDR-GNB carriage upon ICU admission.

Risk Factor	Crude OR	Adjusted OR (95% CI)	*p* Value
Liver cirrhosis	2.81	6.54 (2.17–19.17)	<0.001 *^1^
Previous MDR-GNB carriage	7.45	5.34 (1.55–16.60)	0.005 *^1^
Digestive surgery in the last year	5.27	2.83 (1.29–5.89)	0.007 *^1^
Length of hospital stay in the last year (days)	1.02	1.01 (1.00–1.03)	0.026 *^1^
Previous gastrointestinal endoscopy	3.63	1.98 (0.92–4.01)	0.067 ^1^
APACHE classification system			0.098 ^2^
Emergency surgical (reference)	1	1	
Non-emergency surgical	1.46	4.10 (1.20–16.71)	
Medical	2.20	5.17 (1.76–19.04)	
Cancer chemotherapy	0.65	0.27 (0.04–1.07)	0.113 ^1^
Previous antibacterial therapy	4.60	1.89 (0.83–4.05)	0.114 ^1^
Sex (female)	1.27	1.32 (0.83–2.08)	0.234 ^1^
Age (years)	1.00	1.00 (0.98–1.01)	0.833 ^1^

APACHE: Acute Physiology and Chronic Health Evaluation, MDR-GNB: multidrug-resistant Gram-negative bacteria, OR: odds ratio. * *p*-value < 0.05 of Wald’s test ^1^ or ANOVA’s test ^2^, when appropriate.

## Data Availability

The datasets used and/or analysed during the current study are available from the corresponding author on reasonable request.

## Supplementary Materials

The following supporting information can be downloaded at: https://www.mdpi.com/article/10.3390/ijerph19031039/s1; Text S1: Culture procedure in the microbiology laboratory; Table S1: Baseline characteristics of incident and prevalent cases; Table S2: Previous antibacterial therapy in cases and controls, by groups of antibacterials; Table S3: STROBE (Strengthening The Reporting of Observational Studies in Epidemiology) Checklist.
