# Supplementary material for: Risk Factors for Multidrug-Resistant Gram-Negative Bacteria Carriage upon Admission to the Intensive Care Unit
Source: Int J Environ Res Public Health. 2022 Jan 18;19(3):1039. doi: 10.3390/ijerph19031039 (PMC8834020; doi:10.3390/ijerph19031039)
Supplement: Supplementary file 1 [file ijerph-19-01039-s001.zip › ijerph-1528866-supplementary.pdf]

Supplementary Materials

Text S1. Culture procedure in the microbiology laboratory.

All stool and rectal swab samples were cultured on blood agar, and the chromogenic cultures: ChromID ESBL agar and ChromID CARBA agar (all media were from bioMérieux, France), according to standard laboratory procedures. Cultures were inoculated upon specimen receipt (24 h per day and 7 days per week). Samples were plated as soon as possible upon arrival in the laboratory on all shifts 7 days per week.

Bacteria isolates were initially identified by routine biochemical reactions and were then confirmed to species level using a matrix-assisted laser desorption/ionisation time-of-flight mass spectrometry (MALDI-TOF/MS) system (Microflex LT Biotyper; Bruker Daltonik GmbH, Bermen, Germany).

Minimum inhibitory concentrations (MICs) of multiple agents were determined using a MicroScan WalkAway system (Beckman Coulter, USA) with Neg Combo 58 panels and Neg Combo 82 panels. Additionally, MICs of carbapenems were determined by reference broth microdilution according to the 2019 European Committee on Antimicrobial Susceptibility Testing (EUCAST) guidelines. MIC results were interpreted according to EUCAST breakpoints.

In the case of obtaining growth of any Enterobacteriaceae in the ChromID CARBA agar medium, the NG-Test CARBA 5 immunochromatography (NG Biotech,Guipry, France) was carried out for an early detection of possible carbapenemases.

For detection of carbapenemase genes, bacterial DNA was extracted using a MagCore Automated Extractor System (RBC Bioscience, Taiwan), and a multiplex PCR was performed to detect blaKPC, blaIMP, blaGES, blaVIM, blaNDM and blaOXA-48 genes . Amplicons (both strands) were sequenced. Multilocus sequence typing (MLST) was performed by PCR and sequencing according to the genotyping scheme of the Institut Pasteur.

The microbiological final report communicated was the antibiotic sensitivity profile obtained and the resistance mechanism detected. Those microorganisms producing ESBL or hyperproducing AmpC, both chromosomal derepressed and plasmid was reported, as well as any microorganism producing carbapenemases. Multiresistant *Pseudomonas* and *Acinetobacter* (specifically: resistant to carbapenems or resistant to 1 or more agents in 3 or more families of antibiotics) was also reported.

Table S1. Baseline characteristics of incident and prevalent cases.

Characteristic	Incident cases (n = 91)	Prevalent cases (n = 11)
Age (years), median (IQR)	63.87 (52.94, 74.10)	60.58 (50.36, 68.67)
Sex		
Female	35 (38.5%)	2 (18.2%)
Male	56 (61.5%)	9 (81.8%)
Stay in congregate settings	0	0
Referring ward		
Cardiology	24 (26.4%)	1 (9.1%)
Cardiovascular surgery	9 (9.9%)	1 (9.1%)
General & Digestive surgery	7 (7.7%)	0
Emergency Department	19 (20.9%)	1 (9.1%)
Hepatology	12 (13.2%)	1 (9.1%)
Pulmonology	4 (4.4%)	4 (36.4%)
Other	16 (17.6%)	3 (27.3%)
APACHE classification system		
Medical	73 (80.2%)	9 (81.8%)
Non-emergency surgical	13 (14.3%)	1 (9.1%)
Emergency surgical	5 (5.5%)	1 (9.1%)
Previous hospital admissions	44 (48.4%)	9 (81.8%)
Previous hospital admissions (number), median (IQR)	1 (1, 2)	3 (2, 3)
Length of hospital stay in the last year (days), median (IQR)	5 (0, 16.50)	16 (10.5, 46)
Previous MDR-GNB carriage	6 (6.6%)	11 (100%)
Cancer chemotherapy	2 (2.2%)	0
Dialysis	1 (1.1%)	0
Previous antibacterial therapy	15 (16.5%)	4 (36.4%)
Previous therapy with third-, fourth- or fifth-generation cephalosporins	2 (2.2%)	2 (18.2%)
Previous therapy with carbapenems	2 (2.2%)	2 (18.2%)
Cystic fibrosis	0	1 (9.1%)
Bronchiectasis	1 (1.1%)	1 (9.1%)
COPD	9 (9.9%)	1 (9.1%)
Chronic ulcers	0	0
Liver cirrhosis	8 (8.8%)	1 (9.1%)
Immunodeficiency	1 (1.1%)	0
Cancer	12 (13.2%)	0
Neutropenia	6 (6.6%)	3 (27.3%)
Type 1 diabetes	2 (2.2%)	0
Type 2 diabetes	21 (23.1%)	0
Pressure ulcers	1 (1.1%)	0
Digestive surgery in the last year	15 (16.5%)	3 (27.3%)
Previous gastrointestinal endoscopy	8 (8.8%)	0
Upper	4 (4.4%)	0
Lower	4 (4.4%)	0
APACHE II score (points), mean (SD)	17.76 (9.57)	18.78 (7.24)
Solid organ transplantation	10 (11.0%)	5 (45.5%)
Hematopoietic stem cell transplantation	0	0
Biliary drainage	1 (1.1%)	0

APACHE: Acute Physiology and Chronic Health Evaluation, COPD: chronic obstructive pulmonary disease, IQR: interquartile range, MDR-GNB: multidrug-resistant Gram-negative bacteria, SD: standard deviation.

Table S2. Previous antibacterial therapy in cases and controls, by groups of antibacterials.

Group of antibacterial agents	Cases N = 91 (6.2%)	Controls N = 1385 (93.8%)	Total N= 1476 (100%)
Aminoglycosides	2 (2.2%)	7 (0.5%)	9 (0.6%)
Carbapenems, including combinations with BLI	2 (2.2%)	6 (0.4%)	8 (0.5%)
Cephalosporins: third-, fourth- and fifth-generation, including combinations with BLI	2 (2.2%)	10 (0.7%)	12 (0.8%)
Cephalosporins: other	0	3 (0.2%)	3 (0.2%)
DHFR inhibitors (trimethoprim)	0	2 (0.1%)	2 (0.1%)
Glycopeptides (vancomycin)	2 (2.2%)	5 (0.4%)	7 (0.5%)
Lincosamides (clindamycin)	0	2 (0.1%)	2 (0.1%)
Lipopeptides (daptomycin)	1 (1.1%)	3 (0.2%)	4 (0.3%)
Macrolides	1 (1.1%)	4 (0.3%)	5 (0.3%)
Oxazolidinones (linezolid)	2 (2.2%)	4 (0.3%)	6 (0.4%)
Penicillins, including combinations with BLI	7 (7.7%)	14 (1.0%)	21 (1.4%)
Polymyxins (colistin)	1 (1.1%)	2 (0.1%)	3 (0.2%)
Quinolones	0	7 (0.5%)	7 (0.5%)
Sulfonamides (sulfamethoxazole)	0	1 (0.1%)	1 (0.1%)

DHFR: dihydrofolate reductase, BLI: beta-lactamase inhibitors.

Table S3. STROBE (Strengthening The Reporting of OBservational Studies in Epidemiology) Checklist.

Section and Item	Item No.	Recommendation	Reported on
Title and Abstract	1	(a) Indicate the study's design with a commonly used term in the title or the abstract.	The study design was specified in the abstract. Pages 1, 2.
		(b) Provide in the abstract an informative and balanced summary of what was done and what was found.	Information on the main results was provided in the abstract. Page 2.
Introduction			
Background/Rationale	2	Explain the scientific background and rationale for the investigation being reported.	The scientific background and interest of the study is included in the introduction. Page 4.
Objectives	3	State specific objectives, including any prespecified hypotheses.	Both hypotheses and objectives are included in the introduction. Pages 4, 5.
Methods			
Study Design	4	Present key elements of study design early in the paper.	The study design is explained in material and methods. Page 5.
Setting	5	Describe the setting, locations, and relevant dates, including periods of recruitment, exposure, follow-up, and data collection.	The setting is included in material and methods. Page 5.
Participants	6	Give the eligibility criteria, and the sources and methods of case ascertainment and control selection. Give the rationale for the choice of cases and controls.	Included in material and methods. Page 6.
Variables	7	Clearly define all outcomes, exposures, predictors, potential confounders, and effect modifiers. Give diagnostic criteria, if applicable.	Included in material and methods. Page 6.
Data Sources/ Measurement	8	For each variable of interest, give sources of data and details of methods of assessment (measurement). Describe comparability of assessment methods if there is more than one group.	Included in material and methods. The same form of collection was used in both groups. Pages 6, 7.
Bias	9	Describe any efforts to address potential sources of bias.	Selection bias: consecutive cases were included<br>Information Bias: Standard and well-defined variables were used. Pages 5, 6,7.
Study Size	10	Explain how the study size was arrived at.	All available consecutive cases were included.
Quantitative Variables	11	Explain how quantitative variables were handled in the analyses. If applicable, describe which groupings were chosen and why.	Quantitative variables were treated as such.
Statistical Methods	12	(a) Describe all statistical methods, including those used to control for confounding.	Included in material and methods. Page 7.
		b) Describe any methods used to examine subgroups and interactions.	Included in material and methods. Page 7.
		(c) Explain how missing data were addressed.	No missing data was found.
		(d) If applicable, explain how matching of cases and controls was addressed.	Not applicable.
		(e) Describe any sensitivity analyses.	Not applicable.
Results			
Participants	13	(a) Report numbers of individuals at each stage of study—eg numbers potentially eligible, examined for eligibility, confirmed eligible, included in the study, completing follow-up, and analysed.	Included in material and methods. Page 6.

		(b) Give reasons for non-participation at each stage.	Not applicable.
		(c) Consider use of a flow diagram.	Included in Results.
Participants	14	(a) Give characteristics of study participants (eg demographic, clinical, social) and information on exposures and potential confounders.<br>(b) Indicate the number of participants with missing data for each variable of interest.	Included in Table I. Page 19<br><br>Not applicable.
Outcome Data	15	Report numbers in each exposure category, or summary measures of exposure.	Included in material and methods. Page 6.
Main Results	16	(a) Give unadjusted estimates and, if applicable, confounder-adjusted estimates and their precision (eg, 95% confidence interval). Make clear which confounders were adjusted for and why they were included.<br>(b) Report category boundaries when continuous variables were categorized.<br>(c) If relevant, consider translating estimates of relative risk into absolute risk for a meaningful time period.	Specified in results. Pages 8,9<br><br>Not applicable.<br><br>Not applicable.
Other Analyses	17	Report other analyses done—eg analyses of subgroups and interactions, and sensitivity analyses.	Specified in results and material and methods. Pages 7,8,9
Discussion			
Key Results	18	Summarize key results with reference to study objectives.	Specified in discussion. Pages 9, 10.
Limitations	19	Discuss limitations of the study, taking into account sources of potential bias or imprecision. Discuss both direction and magnitude of any potential bias.	Included in discussion. Pages 10, 11, 12
Interpretation	20	Give a cautious overall interpretation of results considering objectives, limitations, multiplicity of analyses, results from similar studies, and other relevant evidence.	Included in discussion. Pages 10, 11, 12
Generalizability	21	Discuss the generalizability (external validity) of the study results.	Included in discussion. Page 10,11,12.
Other information			
Funding	22	Give the source of funding and the role of the funders for the present study and, if applicable, for the original study on which the present article is based.	Included in funding. Page 13.
